# Altered Spontaneous Brain Activity Related to Neurologic and Sleep Dysfunction in Children With Obstructive Sleep Apnea Syndrome

**DOI:** 10.3389/fnins.2021.595412

**Published:** 2021-11-15

**Authors:** Jie Bai, Hongwei Wen, Jun Tai, Yun Peng, Hongbin Li, Lin Mei, Tingting Ji, Xiaodan Li, Yanhua Li, Xin Ni, Yue Liu

**Affiliations:** ^1^Department of Radiology, Beijing Children’s Hospital, Capital Medical University, National Center for Children’s Health, Beijing, China; ^2^Key Laboratory of Cognition and Personality, Ministry of Education, Chongqing, China; ^3^School of Psychology, Southwest University, Chongqing, China; ^4^Department of Otolaryngology, Head and Neck Surgery, Children’s Hospital Capital Institute of Pediatrics, Beijing, China; ^5^Department of Otolaryngology, Head and Neck Surgery, Beijing Children’s Hospital, Capital Medical University, National Center for Children’s Health, Beijing, China

**Keywords:** obstructive sleep apnea, children, amplitude of low-frequency fluctuation fractional, regional homogeneity, functional magnetic resonance imaging, resting state, spontaneous activity, cognitive impairment

## Abstract

Childhood obstructive sleep apnea (OSA) is a common chronic sleep-related breathing disorder in children, which leads to growth retardation, neurocognitive impairments, and serious complications. Considering the previous studies about brain structural abnormalities in OSA, in the present study, we aimed to explore the altered spontaneous brain activity among OSA patients, using amplitude of low-frequency fluctuation (ALFF), fractional ALFF (fALFF), and regional homogeneity (ReHo) methods based on resting-state functional magnetic resonance imaging (MRI). Thirty-one untreated OSA children and 33 age-and gender-matched healthy children (HC) were included in this study. Compared with controls, the OSA group showed significant lower ALFF in the right lingual gyrus, decreased fALFF in the left middle frontal gyrus (MFG), but increased fALFF in the left precuneus. Decreased ReHo was found in the left inferior frontal gyrus (orbital part) and left middle frontal gyrus. Notably, the mean fALFF value of left MFG was not only significantly related to multiple sleep parameters but also demonstrated the best performance in ROC curve analysis. These findings revealed OSA children were associated with dysfunctions in the default mode network, the frontal lobe, and the lingual gyrus, which may implicate the underlying neurophysiological mechanisms of intrinsic brain activity. The correlation between the altered spontaneous neuronal activity and the clinical index provides early useful diagnostic biomarkers for OSA children as well.

## Introduction

Obstructive sleep apnea (OSA) is a common chronic sleep-related breathing disorder characterized by partial or complete closure of the upper airway during sleep, which results in recurrent intermittent hypoxia, carbon dioxide retention, and frequent awakening (Chen et al., 2016; Stevens et al., 2020). Nowadays, prevalence of OSA increased strikingly with the increasing population age and obesity. The total prevalence of OSA among children ranges from 1 to 4% (Kirk et al., 2017). Clinically, pediatric OSA always presents with loud and irregular snoring and sleep disorders, in contrast to adults, with a range of different symptoms including growth retardation, enuresis, and behavioral problems, such as attention deficit/hyperactivity (ADHD) disorders (Krysta et al., 2017). Besides, noteworthy neurocognitive impairments also occur in pediatric OSA, involving learning, memory, executive function, visuospatial function, and psychomotor development (Copes and Rosentswieg, 1972; Hamasaki et al., 2007; Krysta et al., 2017), which severely diminished academic performance, social adaptation, and even the quality of life in children. Nevertheless, compared with adults, the scholars have paid little attention to brain abnormalities of pediatric OSA before.

Several previous studies exhibited that cortical thinning or gray matter volume reductions occurred in the superior frontal, ventral medial prefrontal, the superior and lateral parietal cortices, the superior temporal lobe, and the brainstem in OSA children, which arose from delayed neuronal development, damage, or atrophy (Philby et al., 2017; Macey et al., 2018). However, unlike in adults, pediatric OSA observed cortical thickening, including the precentral gyrus, the mid-to-posterior insular cortices, and the right anterior insula cortex in OSA children, which might involve in hypoxia-induced inflammatory changes (Macey et al., 2018). Furthermore, entropy measures based on high-resolution T1-weighted imaging identified early damages of brain tissue integrity in pediatric OSA. The affected brain sites included the prefrontal cortex; corpus callosum; and insular, frontal, temporal, hippocampus, and cerebellar areas, which localized within regulated autonomic, respiratory, and cognitive functions (Kheirandish-Gozal et al., 2018). These findings indicated that pediatric OSA presented extensive injury to the brain structures, which might account for underlying executive and cognitive deficits in children.

Currently, resting-state functional magnetic resonance imaging (rs-fMRI) has been found to be a useful and non-invasive technique for detecting spontaneous neural activity. Using rs-fMRI, several studies have demonstrated that OSA patients have shown noteworthy resting-state functional connectivity (rsFC) deficits, especially in the default mode network (DMN), which plays a role in sustaining brain function in the resting-state (Prilipko et al., 2011; Chen et al., 2018). Zhang et al. (2013) reported that OSA patients showed significantly reduced rsFC within the anterior DMN and bilateral fronto-parietal network but increased rsFC between the posterior cingulate cortex and precuneus within the DMN. Similarly, decreased rsFC was observed in DMN subregions, including the medial prefrontal cortex, anterior cingulate, and posterior cingulate in patients with OSA (Chen et al., 2016). Li et al. (2016) found that patients with OSA displayed a dysfunction of rsFC between the right hippocampus formation and posterior cingulate cortex within the DMN and significant negative correlation with delayed memory. In addition, the frontal lobe as a crucial brain area involved in multiple cognitive functions has been increasingly reported. Yu H. et al. (2019) showed that OSA patients showed significantly increased rsFC between the left dorsal amygdala, the right ventrolateral amygdala, and the left inferior frontal gyrus. Regarding these findings, OSA may be related to the abnormal rsFC between distinct brain areas in DMN, fronto-parietal, and limbic system, whereas which area is more responsible for the observed abnormal connectivity was still unclear. It is meaningful to directly locate the abnormal regional spontaneous neural activity in OSA patients during resting state and their relationships with behavioral performances.

Amplitude of low-frequency fluctuation (ALFF), fractional ALFF (fALFF), and regional homogeneity (ReHo) are three major data-driven measures for quantification of spontaneous neural activity based on BOLD signals. ALFF detects the total power within the range between 0.01 and 0.10 Hz and positively correlates to the alterations of spontaneous neural activity (Zang et al., 2007; Zou et al., 2008). Moreover, fALFF measures the ratio of the specific power spectrum of low frequency to that of the total power in the entire frequency range (Qiu et al., 2019). Both ALFF and fALFF have been proven to exhibit greater test–retest reliability, especially in gray matter (Zuo et al., 2010). Notably, fALFF produces better effects in reducing the physiological noise than ALFF, and it can effectively suppress artifacts in non-specific brain regions, such as the ventricles and the vicinity of blood vessels (Zou et al., 2008). ReHo is a data-driven measure for the local measurement of spontaneous neural activity (Xia et al., 2018), and it can effectively evaluate resting-state brain activity based on the hypothesis that brain activity is more likely to occur in clusters rather than in a single voxel (Zhang et al., 2012). Recently, these methods have wide access to explore brain diseases with potential functional alterations, such as depression (Yu Y. et al., 2019), Alzheimer’s disease (Cheng et al., 2019), Tourette syndrome (Liu et al., 2017), and so on. Accordingly, the combination of the three may provide more detailed information about the intrinsic activity than each method alone.

In the present study, we not only investigated abnormal intensity of neural activity via ALFF/fALFF analysis but also investigated abnormal neural synchronization via ReHo analysis in OSA children. Based on previous studies, we hypothesized that (1) OSA children would show altered ALFF/fALFF and ReHo values in the DMN, frontal lobe, and lingual gyrus; (2) the alterations of the spontaneous brain activity would be related to sleep-related respiratory parameters in OSA children; and (3) abnormal spontaneous activity pattern might be utilized as diagnostic neuroimaging biomarkers to discriminate OSA from controls. We aim to take a crucial step towards identifying spontaneous brain activity abnormalities in OSA children and providing potential targets for better understanding and treatment of this neurologic and sleep dysfunction disorder.

## Materials and Methods

### Subjects

Thirty-one OSA children (age: 5.65 ± 2.82 years, range: 3–10 years; 12 female) were recruited in Beijing Children’s Hospital from April 2016 to October 2019. We also included 33 age- (*p* = 0.585, two-sample *t*-test) and gender-matched (*p* = 0.431, chi-square test) healthy children (age: 6.01 ± 2.43 years; range: 2–11 years; 16 female) in our study (Table 1). All the participants were right-handed. The exclusion criteria included (1) history of brain structural injury, neurological or psychiatric disorders; (2) suffering from cardiovascular diseases, neuromuscular diseases, or defined genetic syndromes; (3) abnormal blood pressure, blood fat, and glucose; (4) being with any known acute or chronic illness; and (5) undergone treatment with drugs and surgery. Before the scan, children under 7 years old needed to take chloral hydrate for sedation. The dosage was 0.5 ml/kg and the maximum dose was 10 ml. This study was approved by the Medical Ethics Committee of Beijing Children’s Hospital, Beijing, China. The study was carried out in line with relevant guidelines by the Medical Ethics Committee of Beijing Children’s Hospital, which include MRI scan and clinical diagnosis.

**TABLE 1:** Demographic and clinical characteristics of OSA patients and healthy controls.

	**Characteristics**		**OSA (*n* = 31)**	**HC (*n* = 33)**	***p*-value**
	Gender		19M/12F	17M/16F	0.431^χ2^
	Age (y)		5.65 ± 2.82	6.01 ± 2.43	0.585^*t*^
	Weight (kg)		29.14 ± 18.49	30.05 ± 13.59	0.842^*t*^
	Duration of disease (y)		1.79 ± 1.07	–	–
	BMI (kg/mm^2^)		18.39 ± 5.05	18.40 ± 3.33	0.996^*t*^
	AHI (per hour)		12.90 ± 13.89	1.34 ± 1.30	<0.001^*t*^
	OAI (per hour)		2.30 ± 3.47	0.07 ± 0.15	0.002^*t*^
	HI (per hour)		8.31 ± 11.05	0.69 ± 0.77	0.001^*t*^
	LSaO_2_ (%)		87.82 ± 6.76	93.49 ± 2.84	<0.001^*t*^
	SaO_2_ < 90% (%)		1.31 ± 2.45	0.00 ± 0.00	0.002^*t*^
	Sleep efficiency (%)		83.02 ± 10.28	89.03 ± 8.91	0.016^*t*^
	AI (per hour)		4.89 ± 6.26	0.74 ± 0.83	0.002^*t*^
WISC-V		IQ	97.08 ± 7.69	103.27 ± 15.23	0.229^*t*^
		VIQ	96.00 ± 6.90	106.09 ± 14.75	0.052^*t*^
		PIQ	98.38 ± 10.25	99.18 ± 16.42	0.869^*t*^
	SAFE		9.80 ± 0.41	9.73 ± 0.47	0.656^*t*^
	Attention		17.08 ± 6.50	13.54 ± 9.24	0.233^*t*^
Gesell		Adaptability DQ	80.86 ± 6.15	–	–
Developmental		GMQ	88.14 ± 6.34	–	–
Scale		FMQ	95.43 ± 8.98	–	–
		Language DQ	85.29 ± 11.32	–	–
		Personal-social DQ	88.43 ± 7.87	–	–
		Overall Score	87.71 ± 5.59	–	–
	FD_Jenkinson		0.06 ± 0.05	0.09 ± 0.06	0.065^*t*^

*SaO_2_ < 90%, percentage of total sleep time spent at an oxygen saturation < 90%; LSaO_2_, lowest oxygen saturation; AHI, apnea–hypopnea index; OAI, obstructive apnea index; HI, hypopnea index; AI, arousal index; BMI, body mass index; WISC, the Wechsler Intelligence Scale for Children; VIQ, verbal intelligence quotient; PIQ, performance intelligence quotient; SAFE, Social Adaptive Functioning Evaluation Scale; DQ, developmental quotient; GMQ, gross motor quotient; FMQ, fine motor quotient; t, two-sample t-test; χ^2^, chi-square test. Data are presented as mean ± standard deviation.*

### Polysomnography

All subjects underwent a polysomnography (PSG) evaluation (Compumedics E; Compumedics, Melbourne, Australia; or ALICE 5; Philips Respironics, Amsterdam, Netherlands), which recorded a polysomnogram of more than 7.5 h. Simultaneous monitoring was included for EEG, bilateral electro-oculogram, electromyogram of mentalis activity and bilateral anterior tibialis, ECG, arterial oxyhemoglobin saturation and plethysmographic signal by pulse oximetry, heat-sensitive airflow and nasal pressure, chest and abdominal movements, snoring sensor, body position, and other indicators. All sleep monitoring results were scored manually by experienced professional pediatric PSG technicians according to the diagnostic criteria published by the American Academy of Sleep Medicine (AASM) (Berry et al., 2012). The criteria for the OSA group diagnosis consisted of obstructive apnea (OAI) > 1 times/h or apnea–hypopnea index (AHI) > 5 times/h and lowest oxygen saturation (LSaO_2_) < 92% by PSG.

### Image Acquisition

Magnetic resonance imaging scanning was performed on a 3-T MR scanner (GE Medical Systems, Discovery MR750). Before the scan, all participants should keep respiration and heart rate in a normal state. All participants were required to be awake and quietly breathing until the end of the scan. The scanner parameters for fMRI data are TR/TE = 2000/24 ms, 240 time points, image matrix = 64 × 64, voxel size = 3.5 mm × 3.5 mm × 3.5 mm, field of view (FOV) = 224 mm × 224 mm. The scanner parameters for T1-weighted images are TR/TE = 8.19/3.78 ms, voxel size = 0.4688 mm × 0.4688 mm × 1 mm, matrix = 512 × 512, FOV = 240 mm × 240 mm. During MRI scanning, participants were asked to close their eyes and lie still in the scanner. Head positioning was standardized using canthomeatal landmarks. The head was stabilized with foam pads to minimize its movement.

### Data Preprocessing

Resting-state fMRI data reprocessing was performed using the statistical parametric mapping (SPM8) and Data Processing & Analysis for Resting-state Brain Imaging (DPABI Version 2.1)^1^. The first 10 image volumes of functional images were removed for the signal equilibrium and subject’s adaptation to the scanning noise. Then, the functional images were corrected for time offsets between slices and geometrical displacements due to head motion. We further calculated the mean frame-wise displacement (FD) to measure voxel-wise differences in motion in its derivation (Jenkinson et al., 2002). None of the participants were excluded based on the excluding criteria of 3.0 mm and 3.0 degree in max head motion, with mean FD > 0.2 mm. The T1-weighted images were co-registered to the average functional images and then segmented into the white matter (WM), gray matter (GM), and cerebrospinal fluid (CSF) by using the New Segment tool in DPABI. We removed linear trends and regressed out several nuisance signals from each voxel’s time course, including 24-parameter head-motion profiles (Friston et al., 1996; Yan et al., 2013), mean WM, and cerebrospinal fluid (CSF) time series within the respective brain masks derived from prior probability maps in SPM8 (threshold = 0.8). All the corrected functional data were then normalized by *DARTEL* (Ashburner, 2007) to the Montreal Neurological Institute (MNI) space using an optimum 12-parameter affine transformation and non-linear deformations and then resampled to a 3-mm isotropic resolution.

### Measurement of Amplitude of Low-Frequency Fluctuation/Fractional ALFF and Regional Homogeneity

To calculate ALFF, we firstly performed the spatial smoothing on the resampled images with a 4-mm full width at half maximum (FWHM) Gaussian kernel. We then converted the smoothed signal of each voxel from time domain to frequency domain via Fast Fourier Transform (FFT) to obtain the power spectrum. This power spectrum (frequency range: 0–0.25 Hz) was square-rooted at each frequency, and then averaged across 0.01–0.08 Hz at each voxel, which was taken as ALFF (Zang et al., 2007). To calculate fALFF, the sum of the amplitude (square root of power spectrum) across 0.01–0.08 Hz was divided by that of the entire frequency range (0–0.25 Hz) (Zou et al., 2008). ALFF/fALFF of each voxel was divided by the global mean ALFF/fALFF for standardization purpose, and mALFF/mfALFF was obtained as a parameter for further statistical comparison and analysis.

Regional homogeneity maps were generated before spatial smoothing. After normalization, the band-pass filtering (0.01–0.08 Hz) was performed on the normalized images to reduce the effects of low-frequency drift and high-frequency physiological noise. ReHo maps were conducted by calculating the Kendall coefficient of concordance (KCC) as synchronization of fMRI signals of nearest neighboring 27 voxels (Zang et al., 2004). For standardization purposes, the ReHo value of each voxel was divided by the whole brain mean ReHo value, and then smoothing was done with a 4 mm FWHM Gaussian kernel. The smReHo map was obtained as the ReHo parameter for further statistical comparison and analysis.

### Receiver Operating Characteristic Curves Analysis

Once significantly altered ALFF/fALFF/ReHo areas were found between groups, they might be utilized as markers to discriminate OSA from controls, as useful diagnostic neuroimaging biomarkers (Li et al., 2015). To test this possibility, the mean ALFF/fALFF/ReHo values of significantly altered brain clusters were extracted and used for analysis of the receiver operating characteristic (ROC) curves, using the MedCalc Statistical Software^2^. To summarize the overall diagnostic ability of the tests, we computed the maximum Youden index (sensitivity + specificity − 1) (Fluss et al., 2005), and corresponding sensitivity, specificity, and 95% confidence intervals (CIs) for each cluster.

### Statistical Analysis

Further statistical analysis was performed based on a 90% group mask (meaning 90% of subjects have this voxel) generated in *DPABI* toolbox to detect group differences. We conducted a two-sample *t*-test to compare whole-brain ALFF, ALFF and ReHo values between OSA children and controls, including age, gender, and mean FD (Jenkinson et al., 2002) as covariates. The Gaussian random field (GRF) correction (Bansal and Peterson, 2018) was used to correct for multiple comparisons, and the statistical threshold was set at a voxel level of *p* < 0.001 with a cluster-level *p* < 0.05 (two-tailed) in *DPABI* toolbox. All coordinates were reported in MNI space. Brain regions with significant intergroup differences in ALFF/fALFF/ReHo were defined as regions of interest (ROIs). We extracted the mean ALFF/fALFF/ReHo values of these ROIs from OSA children. Partial correlations controlling for age, gender, and FD values were applied in SPSS version 24.0 (SPSS Inc., Chicago, IL, United States) to identify mean ALFF/fALFF/ReHo values related to clinical parameters of OSAS children, using the statistical threshold of *p* < 0.05.

## Results

### Demographic and Clinical Characteristic

Demographic and clinical characteristics of each group are summarized in Table 1. The OSA had significantly higher scores for AHI, OAI, HI, SaO_2_ < 90%, and AI and significantly lower scores for LSaO_2_ and sleep efficiency than controls. No significant differences were found in age, gender, weight, BMI, and mean FD between the two groups. Of note, there is no significant Pearson correlation between mean FD and age for the OSA group (*r* = 0.197, *p* = 0.289) and control group (*r* = 0.204, *p* = 0.255).

### Amplitude of Low-Frequency Fluctuation Results

**FIGURE 1:**
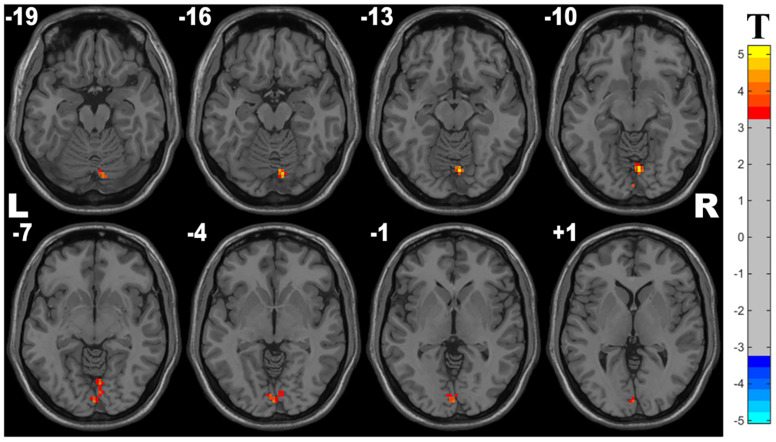
Cluster with significantly decreased ALFF in OSA children compared with controls (with GRF correction, voxel level *p* < 0.001, cluster level *p* < 0.05).

Compared with controls, the OSA group showed significant lower ALFF in the cluster of the right lingual gyrus (Brodmann area 18). The details are presented in Table 2 and Figure 1.

**TABLE 2:** Two-sample *t*-tests demonstrated regions with significantly decreased ALFF in OSA children compared with controls (with GRF correction, voxel level *p* < 0.001, cluster level *p* < 0.05).

**Condition**	**Brain regions**	**Cluster size**	***t*-score of peak voxel**	**MNI coordinates of peak voxel**		
				**x**	**y**	**z**
OSA < HC	Right lingual gyrus (BA 18)	64	5.25	6	−72	−9

*HC, healthy children; BA, Brodmann area.*

### Fractional ALFF Results

**FIGURE 2:**
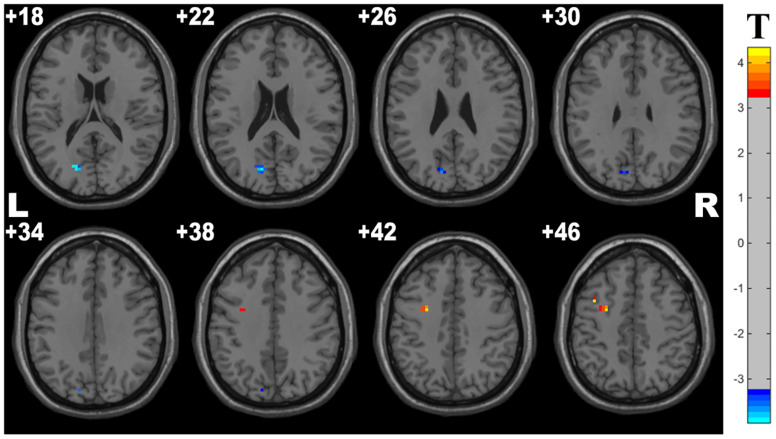
Cluster with significantly altered fALFF in OSA children compared with controls (with GRF correction, voxel level *p* < 0.001, cluster level *p* < 0.05). The blue and red areas denote higher and lower fALFF in OSA group.

Compared with controls, the OSA group showed significant lower fALFF in the cluster of the left middle frontal gyrus and higher fALFF in the left precuneus. The details are presented in Table 3 and Figure 2.

**TABLE 3:** Two-sample *t*-tests demonstrated regions with significantly altered fALFF in OSA children compared with controls (with GRF correction, voxel level *p* < 0.001, cluster level *p* < 0.05).

**Condition**	**Brain regions**	**Cluster size**	***t*-score of peak voxel**	**MNI coordinates of peak voxel**		
				**x**	**y**	**z**
OSA > HC	Left precuneus	32	3.98	−18	−75	18
OSA < HC	Left middle frontal gyrus	22	4.34	−27	0	45

### Regional Homogeneity Results

**FIGURE 3:**
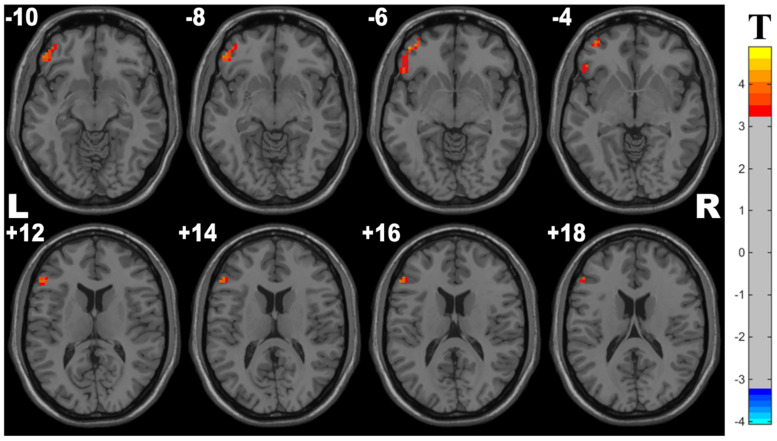
Cluster with significantly decreased ReHo in OSA children compared with controls (with GRF correction, voxel level *p* < 0.001, cluster level *p* < 0.05).

Compared with controls, OSA group showed significant lower ReHo in the cluster of the left inferior frontal gyrus (orbital part) and left middle frontal gyrus. The details are presented in Table 4 and Figure 3.

**TABLE 4:** Two-sample *t*-tests demonstrated regions with significantly decreased ReHo in OSA children compared with controls (with GRF correction, voxel level *p* < 0.001, cluster level *p* < 0.05).

**Condition**	**Brain regions**	**Cluster size**	***t*-score of peak voxel**	**MNI coordinates of peak voxel**		
				**x**	**y**	**z**
OSA < HC	Left inferior frontal gyrus, orbital part	50	4.15	−45	48	−6
OSA < HC	Left middle frontal gyrus	58	4.39	−45	33	27

### Receiver Operating Characteristic Curves Analysis Results

**FIGURE 4:**
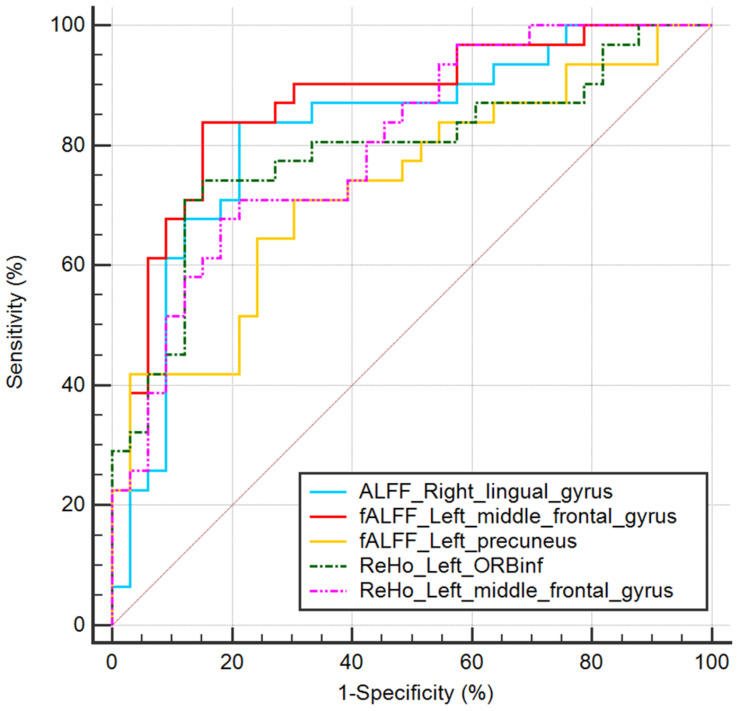
ROC curve analysis of mean ALFF/fALFF/ReHo signal values for altered brain clusters. AUC, area under the curve; ORBinf, inferior frontal gyrus, orbital part.

In the ROC curve analysis, the mean fALFF value of left middle frontal gyrus achieves the highest sensitivity, specificity, and area under the ROC curve (AUC). All the altered brain clusters achieve the significance level *p* < 0.001 of AUC, indicating these findings as potential useful diagnostic biomarkers. The details are presented in Table 5 and Figure 4.

**TABLE 5:** The statistics of ROC curve analysis for altered brain clusters.

**Clusters**	**SEN**	**SPE**	**AUC**	**95% CIs**
ALFF_Right lingual gyrus	83.87%	78.79%	0.822	0.706–0.906
fALFF_Left precuneus	70.97%	69.70%	0.732	0.607–0.835
fALFF_Left middle frontal gyrus	83.87%	84.85%	0.871	0.764–0.942
ReHo_Left ORBinf	74.19%	84.85%	0.790	0.670–0.882
ReHo_Left middle frontal gyrus	70.97%	78.79%	0.802	0.683–0.891

*SEN/SPE, sensitivity/specificity corresponding to maximum Youden index; AUC, area under the ROC curve; CIs, confidence intervals; ORBinf, inferior frontal gyrus, orbital part.*

### Correlation Results

**FIGURE 5:**
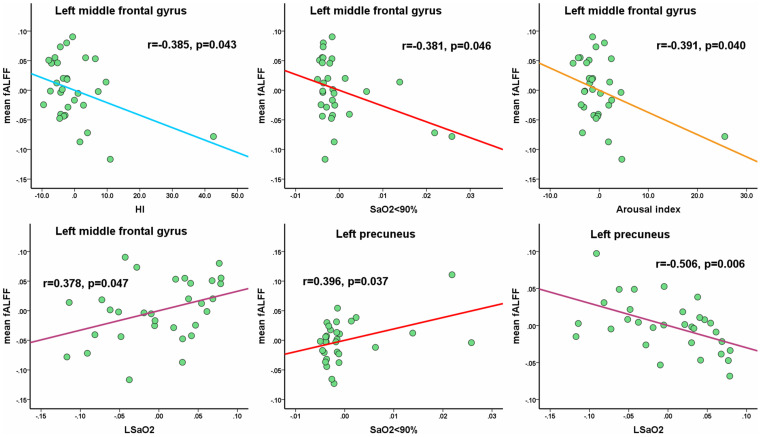
The mean fALFF values in altered clusters show the significantly partial correlations with clinical parameters. Of note, the coordinate value of both *X*-axis (clinical parameter) and *Y*-axis (fALFF value) do not reflect the initial values of these variables, while considering age, gender and FD as covariates. All the *p*-values were uncorrected for multiple comparisons.

In the OSA group, the mean fALFF value in the left middle frontal gyrus showed significantly negative correlations with HI (*r* = −0.385, *p* = 0.043), SaO_2_ < 90% (*r* = −0.381, *p* = 0.046), AI (*r* = −0.391, *p* = 0.040), and a significantly positive correlation with LSaO_2_ (*r* = 0.378, *p* = 0.047). The mean fALFF value in the left precuneus showed a significantly negative correlation with LSaO_2_ (*r* = −0.506, *p* = 0.006) and a significantly positive correlation with SaO_2_ < 90% (*r* = 0.396, *p* = 0.037) (shown in Figure 5). All the p-values were uncorrected for multiple comparisons.

## Discussion

Amplitude of Low-Frequency Fluctuation/Fractional ALFF and ReHo approaches are used to investigate the intrinsic brain activities in OSA, as effective noninvasive imaging tools. These methods are based on different neurophysiological mechanisms, ALFF/fALFF analysis represents neural intensity, whereas ReHo reflects neural coherence (Liu et al., 2017). Our study is the first to utilize the combination of three methods to detect abnormal neural activities in certain brain regions in pediatric OSA, which contributed to the exhibition of more comprehensive functional alterations. Compared to previous study (Li et al., 2015), we applied more clinical parameters analysis and obtained more outstanding correlations with functional parameters. Moreover, the ROC curve analysis was demonstrated in detail, along with sensitivity and specificity, which provided remarkable guidance for clinical diagnosis. In addition, a large sample size was enrolled with 31 OSA individuals in this study. Taken together, we believe our study contributes to obtain more reliable results for revealing the abnormalities of neural activity in OSA children.

Compared with healthy controls, the OSA group showed significant lower ALFF in the right lingual gyrus (Brodmann area 18). In the OSA group, fALFF was significantly decreased in the left middle frontal gyrus as well as increased in the left precuneus. We also observed that the OSA group showed a significant decrease of ReHo in the left inferior frontal gyrus (orbital part) and left middle frontal gyrus. These results revealed the changes of spontaneous brain activities were closely associated with the DMN, the frontal lobe, and the right lingual gyrus. Furthermore, in the OSA group, the mean fALFF value in the left middle frontal gyrus showed significantly negative correlations with HI, SaO_2_ < 90%, and AI and a significantly positive correlation with LSaO_2_. The mean fALFF value in the left precuneus showed a significantly negative correlation with LSaO_2_ and a significantly positive correlation with SaO_2_ < 90%. Additionally, in the previous studies (Zang et al., 2007; Zou et al., 2008), scholars have found the ALFF method may be affected by physiological noise irrelevant to brain activity. However, the artifacts from non-specific brain areas (the ventricles and the vicinity of blood vessels) were significantly reduced by the fALFF approach, while signals from cortical regions correlated with brain activity were enhanced, which contributed to the superior sensitivity and specificity in detecting spontaneous brain activities (Zou et al., 2008). In line with previous researches, in our study, we also noted the fALFF approach showed more rewarding results among these approaches. The mean fALFF value not only significantly associated with the sleep parameters but also demonstrated the best performance in ROC curve analysis.

Our study revealed the increase of fALFF in the left precuneus. The precuneus is located in the posteromedial cortex of the parietal lobe and plays a key role in a range of highly integrated tasks, including visuospatial imagery, self-processing operations, and episodic memory retrieval, namely, first-person perspective taking and an experience of agency (Cavanna and Trimble, 2006). Peng et al. (2014) found that patients with OSA showed decreased ReHo in the precuneus, besides the significant negative correlation between altered ReHo in the precuneus with sleep time, suggesting decreased sleep time might be a key factor for dysfunction in the precuneus. An 18F-fluoro-2-deoxy-D-Glucose positron emission tomography study showed a decrease of brain metabolism in the precuneus (Yaouhi et al., 2009). Another functional imaging study displayed lower ALFF in the right precuneus; in addition, a significant positive correlation was found in the right precuneus between MoCA score and the ALFF value, which indicated the abnormalities of the precuneus may be associated with a cognitive dysfunction (Li et al., 2015).

As is well known, the precuneus is a vital node in the default mode networks (DMN), and others include the posterior cingulate cortex; medial prefrontal cortex; and medial, lateral, and inferior parietal regions. These brain regions are highly interconnected to form DMN, a large-scale network, which is thought to be involved in an array of advanced cognitive functions, such as visuospatial imagery, consciousness, attention, adaptation, episodic memory, executive cognitive control, and behavioral inhibition (Andrews-Hanna et al., 2010; Wilson et al., 2010). As similarly reported in the past, abnormal inactivation in the DMN among patients with OSA was found during working memory tasks and also significantly positively correlated with behavioral performance, which may imply that the inhibition of activity in the DMN plays a role in cognitive impairment (Prilipko et al., 2011). Specifically, reduced functional connectivity is related to cognitive dysfunction in the DMN at rest and the enhanced functional connectivity of the OSA may be a compensatory mechanism for the decrease of cognition (Chen et al., 2016). Chen et al. (2018) found that the OSA group showed significantly decreased FC of the anterior–posterior DMN and within the posterior DMN and was associated with the MoCA score, using graph theory approaches. Furthermore, they found abnormal FC within the DMN may contribute to the topological reconfiguration of the DMN in patients with OSA, which illuminated the cognitive dysfunction and topological reconfiguration in OSA. Nevertheless, based on these findings, we found that patients with OSA had increased fALFF in the left precuneus, which may imply an adaptive compensatory response in the DMN. Furthermore, our study also found a significant negative correlation between the altered fALFF in the local region of the DMN and the LSaO_2_, suggesting that intermittent hypoxia may be a principal element for the DMN abnormity in OSA.

Our study showed fALFF and ReHo were decreased in the left middle frontal gyrus and the left inferior frontal gyrus (orbital part), and left middle frontal gyrus, respectively. To our knowledge, the frontal lobe is the higher cortex of cognitive executive function and also plays an important role in emotion, language, attention, working memory, problem solving, impulse control, and social behavior (Stern et al., 2001; Johnson et al., 2009; Nie et al., 2017). Huang et al. (2019) found both decreased gray matter volume (GMV) and functional response in the orbital frontal cortex (OFC) in patients with OSA, which may indicate that OFC is a vulnerable and sensitive area in the brain. Another study demonstrated that patients with OSA showed significantly lower ReHo in the right medial frontal gyrus and right superior frontal gyrus (Peng et al., 2014). Ji et al. (2021) also found significant lower ReHo in the left medial superior frontal gyrus and was positively associated with VIQ, which reflected that such brain area may play a crucial role in the cognitive processing related to VIQ. The inferior frontal gyrus was attributed to the language function, decision making under risk, and the regulation of cognitive control (Foland-Ross and Gotlib, 2012; Luo et al., 2015). Song et al. (2018) found that the impaired FC between the caudate and inferior frontal gyrus may become the basis of the cognitive regulation defects of emotion, which leads to comorbid mood disorders in OSA.

Consistent with these findings, in our study, we found that patients with OSA showed significantly altered brain activities in the left frontal gyrus when compared with controls. Notably, the mean fALFF value of left middle frontal gyrus achieves the highest sensitivity, specificity, and AUC value in ROC curve analysis. Also, significant correlations were found between the mean fALFF values of the left middle frontal gyrus with the more clinical parameters, suggesting the middle frontal gyrus might be a sensitive region in the brain among OSA children, which might turn into the potential useful diagnostic biomarkers.

In the present study, we also observed significant lower ALFF in the right lingual gyrus among patients with OSA. The lingual gyrus of the occipital lobe is located between the calcarine sulcus and the posterior portion of the collateral sulcus and then extends to the tentorial surface of the temporal lobe and joins the hippocampus (Joo et al., 2007). A study by Luo et al. (2015) found that the OSA showed a tendency of decreased degree in the right lingual gyrus, as a topological alteration in and regional properties in patients with OSA. Our study is also consistent with their results of different modalities. Besides, Joo et al. (2007) found that the cerebral blood flow of parahippocampal and lingual gyrus were reduced in OSA during wakefulness, using the 99mTc-ethyl cysteinate dimer (ECD) single photon emission computed tomography method, which partly indicated memory impairment and spatial learning deficits in patients with severe OSA. The lingual gyrus has been believed to be involved in visual recognition and episodic memory consolidation (Kukolja et al., 2016) and are considered to play a role in the process of generating and recalling dreams as well (Bischof and Bassetti, 2004). Therefore, the decreased metrics of lingual gyrus possibly account for certain deficits in visual memory and learning.

## Limitations

Several limitations deserve to be mentioned in the present study. Firstly, our study obtained a larger sample size compared with previous neuroimaging researches in pediatric OSA (Luo et al., 2015; Ji et al., 2021). However, the sample size was still relatively small, which implies an urgent need for expanding data to validate the results of future studies. Secondly, the *p*-values of the correlations between network properties and clinical measurements are not corrected for multiple comparisons in this study, as this is currently a preliminary exploratory research. We will reveal the correlations corrected for multiple comparisons based on more subjects in future research, which obtains the more reliable mechanisms of brain activity in OSA children. Thirdly, the previous study found that the test–retest reliability of PerAF is better than ALFF and fALFF, and the test–retest reliability between machines is better (Zhao et al., 2018). We will investigate this using perAF to measure the spontaneous brain activity of OSA children in our future study.

## Conclusion

We have investigated the alterations of spontaneous neural activity in OSA, based on the ALFF/fALFF and ReHo approaches on rs-fMRI data. Abnormal regions with the altered neural activity in OSA children include the precuneus, the middle and inferior frontal gyrus, and the lingual gyrus. Moreover, we also found that the altered fALFF in the precuneus was negatively correlated with the LSaO_2_ in OSA. These results expounded the underlying neurophysiological mechanisms of altered spontaneous brain activity and revealed the correlation between the changes of sleep function and functional activity in brain.

## Data Availability Statement

The original contributions presented in the study are included in the article/supplementary material, further inquiries can be directed to the corresponding author/s.

## Ethics Statement

The studies involving human participants were reviewed and approved by the Medical Ethics Committee of Beijing Children’s Hospital, Beijing, China. Written informed consent to participate in this study was provided by the participants’ legal guardian/next of kin.

## Author Contributions

JB: study concept and design, investigation, acquisition of data, analysis and interpretation of data, and drafting of the original manuscript. HW: study concept and design, methodology, software, analysis and interpretation of data, and writing – review and editing. JT: collect clinical information and access cognitive function. YP and XN: study supervision and project administration. HL, LM, TJ, and XL: collect clinical information and access cognitive function. YaL: acquisition of data. YuL: study concept and design, and writing – review and editing. All authors contributed to the article and approved the submitted version.

## Conflict of Interest

The authors declare that the research was conducted in the absence of any commercial or financial relationships that could be construed as a potential conflict of interest.

## Publisher’s Note

All claims expressed in this article are solely those of the authors and do not necessarily represent those of their affiliated organizations, or those of the publisher, the editors and the reviewers. Any product that may be evaluated in this article, or claim that may be made by its manufacturer, is not guaranteed or endorsed by the publisher.

## Footnotes

1
http://www.restfmri.net

2
https://www.medcalc.org
